# Inferring ecological explanations for biogeographic boundaries of parapatric Asian mountain frogs

**DOI:** 10.1186/s12898-018-0160-5

**Published:** 2018-02-02

**Authors:** Junhua Hu, Jianping Jiang

**Affiliations:** 1 Key Laboratory of Southwest China Wildlife Resource Conservation (China West Normal University), Ministry of Education, Nanchong, 637009 China; 2 Chengdu Institute of Biology, Chinese Academy of Sciences, Chengdu, 610041 China

**Keywords:** Amphibians, Contact zone, Ecological niche models, Ecological barrier, Interspecific competition, Niche conservatism, Niche overlap, Qinling Mountains

## Abstract

**Background:**

Identifying and understanding the mechanisms that shape barriers to dispersal and resulting biogeographic boundaries has been a longstanding, yet challenging, goal in ecology, evolution and biogeography. Characterized by stable, adjacent ranges, without any intervening physical barriers, and limited, if any, range overlap in a narrow contact zone, parapatric species are an interesting system for studying biogeographic boundaries. The geographic ranges of two parapatric frog species, *Feirana quadranus* and *F. taihangnica*, meet in a contact zone within the Qinling Mountains, an important watershed for East Asia. To identify possible ecological determinants of the parapatric range boundaries for two closely related frog species, we quantified the extent of their niche differentiation in both geographical and environmental space combining ecological niche models with an ordination technique. We tested two alternative null hypotheses (sharp environmental gradients versus a ribbon of unsuitable habitat dividing two highly suitable regions) for biogeographic boundaries, against the null expectation that environmental variation across a given boundary is no greater than expected by chance.

**Results:**

We found that the niches of these two parapatric species are more similar than expected by chance, but not equivalent. No sharp environmental gradient was found, while a ribbon of unsuitable habitat did act as a barrier for *F. quadranus*, but not for *F. taihangnica*.

**Conclusions:**

Integrating our findings with historical biogeographic information, our results suggest that at a contact zone, environmental tolerance restricted *F. quadranus* from dispersing further north, while interspecific competition most likely prevented the southward expansion of *F. taihangnica*. This study highlights the importance of both climate and competition in exploring ecological explanations for parapatric range boundaries between ecologically similar frog species, in particular under the effects of changing climate.

**Electronic supplementary material:**

The online version of this article (10.1186/s12898-018-0160-5) contains supplementary material, which is available to authorized users.

## Background

Identifying and understanding the mechanisms that shape barriers to dispersal and resulting biogeographic boundaries continue to be a challenge in biogeography, evolution and conservation [1–3]. Numerous ecological and evolutionary factors have been identified to can strongly influence biogeographic boundaries for species (e.g. climate [4], dispersal capacity [1], physical barriers [5], interspecific interactions [6, 7], population dynamics and evolutionary potential [8]). Both abiotic and biotic factors are thought to shape species’ range boundaries, with recent attention focused on the interaction of these factors [9–11]. Resolving the extent to which these factors delimit species’ range boundaries across large spatial scales has been particularly contentious [12–14].

Abiotic factors can not only impose species’ range boundaries directly by causing mortality, or by preventing successful reproduction or completion of the life cycle [15], but also indirectly by causing increases in the number of competitors or predators [16]. Climate is often found to be the dominant abiotic force determining species’ distribution ranges at broad spatial scales [15, 17]. However, interspecific interactions (a biotic factor) can also shape distributions for a large number of species [1, 6, 15], with detectable effects on range boundaries at broad spatial scales [4, 7, 18]. To address the relative strength of abiotic versus biotic factors in limiting species’ distributions, one longstanding macroecological hypothesis, as refined by MacArthur [19] (and termed the north–south hypothesis by Cunningham et al. [11]), suggests that abiotic conditions (particularly climate) determine a species’ poleward range boundary while interspecific interactions delineate the equatorial boundary [12, 15].

The range boundaries of species may be allied with limits to environmental niche expansion [20]. With rapidly expanding geographic information system (GIS)-based environmental data and known occurrences catalogued in biodiversity collections, we can identify ecological barriers to dispersal [21]. Ecological niche models (ENMs) combine spatially explicit environmental data with occurrence data to characterize taxa’s environmental requirements and to predict environmental suitability. ENMs, used in conjunction with recently developed tests of niche overlap, are a useful tool for exploring ecological divergence among taxa and the role of ecological factors in range boundaries for closely related taxa, at large geographic scales (e.g. [4, 7, 22]). Characterized by stable, adjacent ranges, without any intervening physical barriers, and limited, if any, range overlap in a narrow contact zone, parapatric species are an interesting system for studying biogeographic boundaries [4, 7, 8, 18]. Newly available data and methods (e.g. ENMs and associated comparative metrics) may be utilized to provide insight into whether or not contact zones coincide with species’ niche limits [4, 7, 22, 23].

**Fig. 1:**
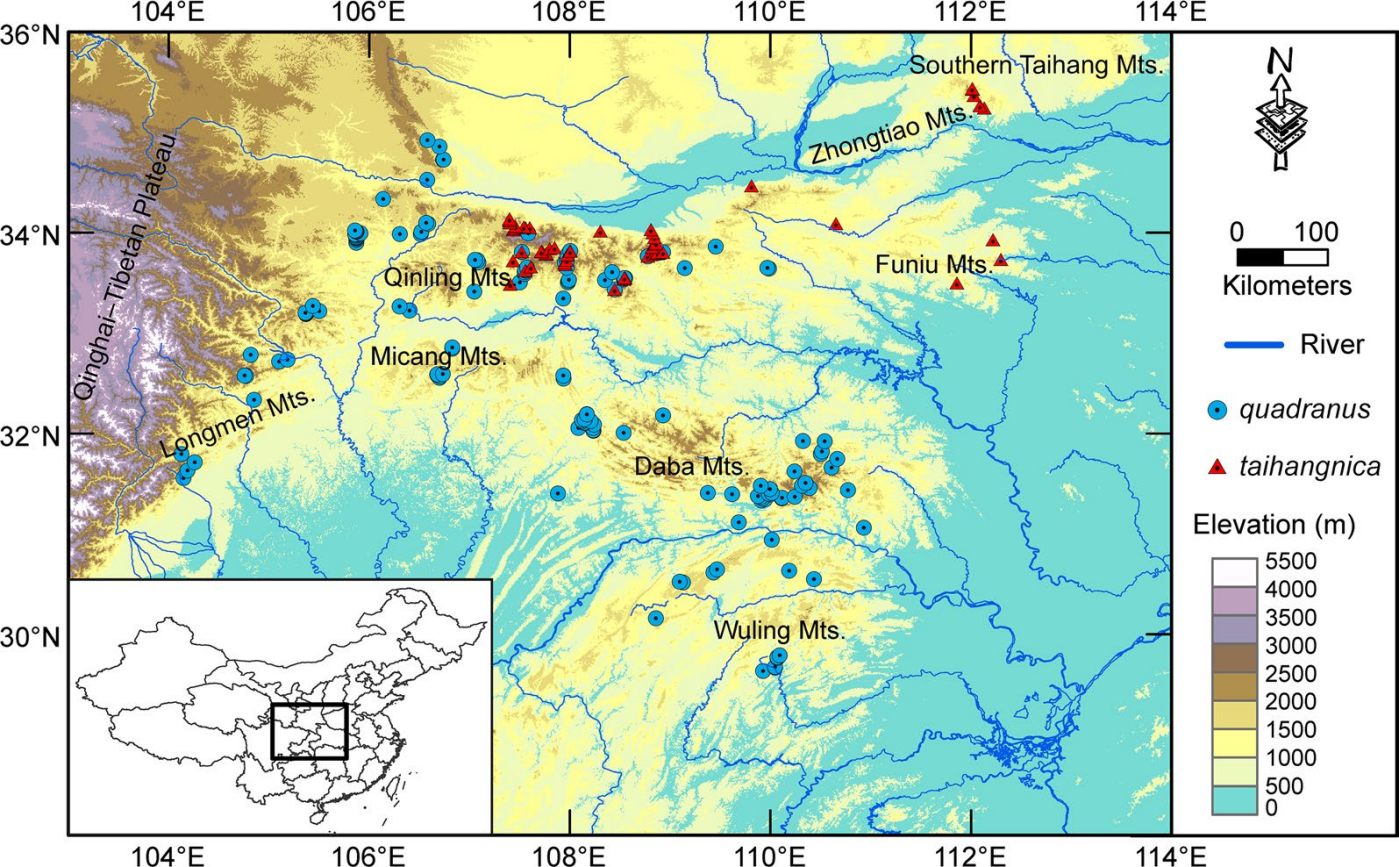
Species’ distributions based on occurrence records for *Feirana quadranus* and *F. taihangnica*. The bottom-left inset shows the geographical location of the Qinling-Daba Mountains in mainland China (bold-black rectangle)

Frogs in the genus *Feirana* (family Dicroglossidae; Dubois, 1992) radiated in the Qinling-Daba Mountains (QDM; [24]), with the ancestors of *Feirana quadranus* (hereafter, *quadranus*; swelled-vented frog) and *F. taihangnica* (hereafter, *taihangnica*; Taihangshan swelled-vented frog) subsequently diversifying in the Longmen-Micang-Daba Mountains [25] and the Qinling Mountains [26], respectively. Based on phylogeographic analyses, after the last glacial maximum (LGM), populations of *quadranus* from either the Longmen or Daba Mountains massively invaded the Qinling Mountains, while *taihangnica* substantially expanded its range from the Qinling Mountains to the Zhongtiao-Southern Taihang Mountains [24–26]. These historical range expansions and shifts resulted in the geographical distributions of these two species meeting in the Qinling Mountains [24], an important watershed for East Asia. The present day distribution of *quadranus* extends throughout the QDM while *taihangnica* is endemic to the Qinling Mountains [25, 26], with *quadranus* living in a wider environmental space than *taihangnica* encircled by annual mean temperature and annual precipitation (Additional file 1). A substantial contact zone between the two species is located along the southern range boundary of *taihangnica* in the central Qinling Mountains (Fig. 1; [24]).

Both *quadranus* and *taihangnica* have similar natural histories and closely resemble each other ecologically and phenotypically, without obvious differences in body size or secondary sexual characteristics [24, 27, 28]. However, breeding males do differ in one significant trait: *taihangnica* breeding males possess a multitude of tiny granules on the swollen skin of the anal area. These are important for species recognition and male-male competition, as well as for female mate choice [27, 29]. Anecdotal evidence of interspecific competition has suggested that *taihangnica* may be displaced when co-occurring with *quadranus* [25, 26]. As such, these two parapatric frog species which meet along an elongated contact zone (Fig. 1) seem to present an excellent opportunity for comparing their niche divergence and assessing whether parapatric range boundaries are associated with niche limits. These two species are also well suited for exploring ecological determinants of parapatric range boundaries at broad spatial scales, as they are closely related and similar in morphology and microhabitat selection [24, 28].

Here, we combined ENMs with multivariate approaches, and used measures of niche similarity to quantify the extent of niche differentiation between *quadranus* and *taihangnica*, and to identify significant ecological barriers for their parapatric range boundaries. We first compared species’ Grinnellian niches [10] to identify the degree of ecological differentiation between the species. Next, we explored the question of whether the niches of two species are more similar than expected by chance, but not equivalent, by comparing niches in both environmental (E)-space and geographical (G)-space [30–32]. We then tested if there was a sharp environmental gradient within the contact zone between species. Finally, we tested the hypothesis that a ribbon of unsuitable habitat divided two highly suitable regions, and that this shaped the parapatric range boundaries of these two congeneric frogs.

## Methods

### Species and environmental data, and environmental niche modeling

We gathered occurrence records from field expeditions for both species from June to September of 2010–2014 (see also [25, 26, 28]). These direct field observations were supplemented with occurrence data from georeferenced specimens in the Herpetological Museum of the Chengdu Institute of Biology, to cover the entirety of species’ geographical ranges. Due to the phenotypic similarity and lack of natural hybridization and introgression between the species (determined from analyses of nuclear microsatellites; Wang et al. unpublished data), we obtained mitochondrial ND2 sequences for individuals from all known populations to aid species identifications [24–26, 28]. We assigned individuals from the contact zone to species relying on ND2 sequences [24, 28]; however, we identified individuals from allopatric areas to species mainly using morphological characters and geographic information, with the assistance of ND2 sequences. A total of 1269 georeferenced occurrences were documented. We filtered occurrences spatially to exclude duplicate occurrences within the same grid cell for each species at a spatial resolution of 30 arc-seconds (~ 1 km at the equator) [23]. Our final dataset comprised 144 georeferenced occurrences records for *quadranus* and 56 for *taihangnica* (Fig. 1).

We initially compiled a set of environmental variables to describe environmental heterogeneity (Additional file 2). To avoid the problem of “over-fitting” in modeling, we reduced the number of variables using the results of Pearson’s correlation tests and a jackknife analysis. Specifically, certain temperature variables were removed owing to high correlations with other temperature variables (|*r*| > 0.75) and likewise for precipitation variables. Variables with relatively higher percent contributions were retained in the jackknife analysis [33]. We retained nine variables in the final ENMs including: annual mean temperature (T_*anu*_), mean monthly temperature range (T_*ran*_), temperature seasonality (T_*sea*_), maximum temperature of the warmest month (T_*max*_), minimum temperature of the coldest month (T_*min*_), mean temperature of the coldest quarter (T_*col*_), annual precipitation (Prec_*anu*_), precipitation seasonality (Prec_*sea*_) [34] and annual actual evapotranspiration (AET_*anu*_; http://www.cgiar-csi.org). With a resolution of 30 arc-seconds, these variables reflected meaningful environmental conditions to which frogs are exposed and which are known to impose constraints on the physiology and survival of amphibian species [17, 35].

We used the maximum entropy algorithm in Maxent 3.3.3 k [33] to developed ENMs for *quadranus* and *taihangnica*. Ten cross-validation replicates were generated, with 70% of occurrences used for model training and 30% for testing. The jackknife option in Maxent was used to evaluate the importance of each variable. Other parameters were set to default [36]. The average of the replicates was used for subsequent analyses. To assess model performance, we calculated the average value of the area under the receiver operating characteristic curve (AUC) for training and test datasets [37]; AUC takes on values ranging from 0.5 (no better discrimination than random) to 1 (perfect discrimination). Logistic output format was selected with values ranging from 0 (lowest) to 1 (highest) [36].

Based on the ENMs outputs, we created a binary presence/absence map from the continuous suitability using the 10th percentile training threshold [38]. We extracted the values for each environmental variable within the predicted distributions and carried out a principal component analysis (PCA). We performed an multivariate analysis of variance (MANOVA), with the first two principal components (PCs) as dependent variables and species as a categorical variable, to compare the environmental envelopes of the two species [39].

### Testing whether the niches of two parapatric species are more similar than expected by chance, but not equivalent

To robustly estimate niche differentiation in evolutionary and community contexts, the niche comparisons have been developed by means of both ENMs in G-space and ordination techniques in E-space [30, 32]. We first used two randomization tests introduced by Warren et al. [30] to examine (1) whether the niches of *quadranus* and *taihangnica* were equivalent (ENM-based niche equivalency test), and/or (2) more or less similar than expected by chance, based on their environmental backgrounds (ENM-based randomization test of background similarity). These tests are based on two similarity metrics (*I* and Schoener’s *D*) that compare ENM predictions of habitat suitability for each grid cell in the study area, after normalizing each species’ ENM so that all similarity scores sum to one; values range from 0 (no niche overlap) to 1 (complete overlap). We calculated these metrics in ENMTools v1.3 [40], using 100 replicates to generate a pseudoreplicated null distribution. Observed niche overlap was then compared to the null distributions, for both the niche equivalency and similarity tests. The null hypothesis of niche equivalency is rejected when observed values of *I* or *D* are significantly different from the pseudoreplicated datasets. Using a randomization procedure, we also performed a background similarity test in reciprocal directions for the species pair [40].

Next, we carried out the niche equivalency and similarity tests using the ordination technique of PCA-env in E-space, which can most accurately retrieve the simulated level of niche overlap and without substantial bias [32]. PCA-env calculates the densities for both occurrences and environmental variables along environmental (principal component) axes for each cell using a kernel smoothing method and then uses these densities to measure niche overlap along these axes. Occurrences are then projected onto the gridded E-space (at a resolution of 100  ×  100 cells) of the first two axes for ordinations such as PCA calculated with the environmental variables. An unbiased estimate of the Schoener’s *D* metric can be calculated for our data and is ensured to be independent of the resolution of the grid. Statistical confidence in niche overlaps was then tested through a one-sided niche-similarity test [32]. We used the background defined by a geographic minimum convex polygon (MCP) with a 50-km buffer that circumscribed occurrences for each species [31, 40], in ArcGIS 9.2 (ESRI, Redlands, CA). All statistical analyses were performed in R 3.0.2 [41] using scripts in Broennimann et al. [32].

### Testing ecological explanations for parapatric range boundaries

We tested two alternative ecological explanations for parapatric range boundaries, i.e. an environmental gradient versus a ribbon of unsuitable habitat between two highly suitable regions, by testing whether the boundary between species was associated with significant environmental variation [21]. Firstly, to test whether or not the species’ range boundaries within the contact zone coincided with an abrupt environmental transition, we performed both linear and blob range-breaking tests. For the linear range-breaking test, occurrences of both species were pooled before randomly drawing a line through all occurrences, dividing them into two artificial “species”; ENMs were then generated for each set of occurrences to either side of the line. For the blob range-breaking test, pseudoreplicate, non-linear species’ ranges were generated by randomly selecting a single point (from the pooled occurrences) and then expanding from this point to partition the dataset to match the desired number of occurrences for both species. For both tests, we generated null distributions for the similarity metrics *I* and *D*, by calculating niche overlap in each of 100 random replicates. The null hypothesis, of no environmental transition, is rejected when the observed value of *I* or *D* is lower than 95% of the values in the null distribution [21].

Next, to address whether or not the contact zone between *quadranus* and *taihangnica* represents a ribbon of unsuitable habitat separating areas of high suitability, we used a random ribbon range-breaking test [21]. For this test, we generated ENMs for three groups of occurrences—samples within the contact zone, and allopatric samples at either side of the contact zone—and then measured overlap between all three possible pairs. We also did this for 100 range-break replicates, where a ribbon was drawn randomly through the pooled occurrences of both species. The width of the ribbon was kept constant, and was estimated based on the width of the contact zone (i.e. the zone of co-occurrences between species in Fig. 1, about 50 km; see also Fig. 1 in Wang et al. [24]). By calculating *I* and *D* from these pseudoreplicates, we generated null distributions with which we could address whether the allopatric areas for each species were more environmentally similar to each other than either was to the contact zone.

## Results

The ENMs of both *quadranus* and *taihangnica* were characterized by high AUC statistics (training AUC: *quadranus* = 0.954 ± 0.006 SD, *taihangnica* = 0.978 ± 0.003; test AUC: *quadranus* = 0.922 ± 0.017, *taihangnica* = 0.975 ± 0.016), indicating that these ENMs successfully discriminated real occurrences from background locations. Jackknife tests on variable importance for *quadranus* revealed that T_*min*_ was the highest ranked variable, showing the greatest model quality when used in isolation, while T_*sea*_ produced the greatest decrease in gain when excluded from the model. For *taihangnica*, T_*col*_ was the most important variable when used in isolation, while Prec_*sea*_ decreased the gain the most when excluded (Additional file 3).

**Fig. 2:**
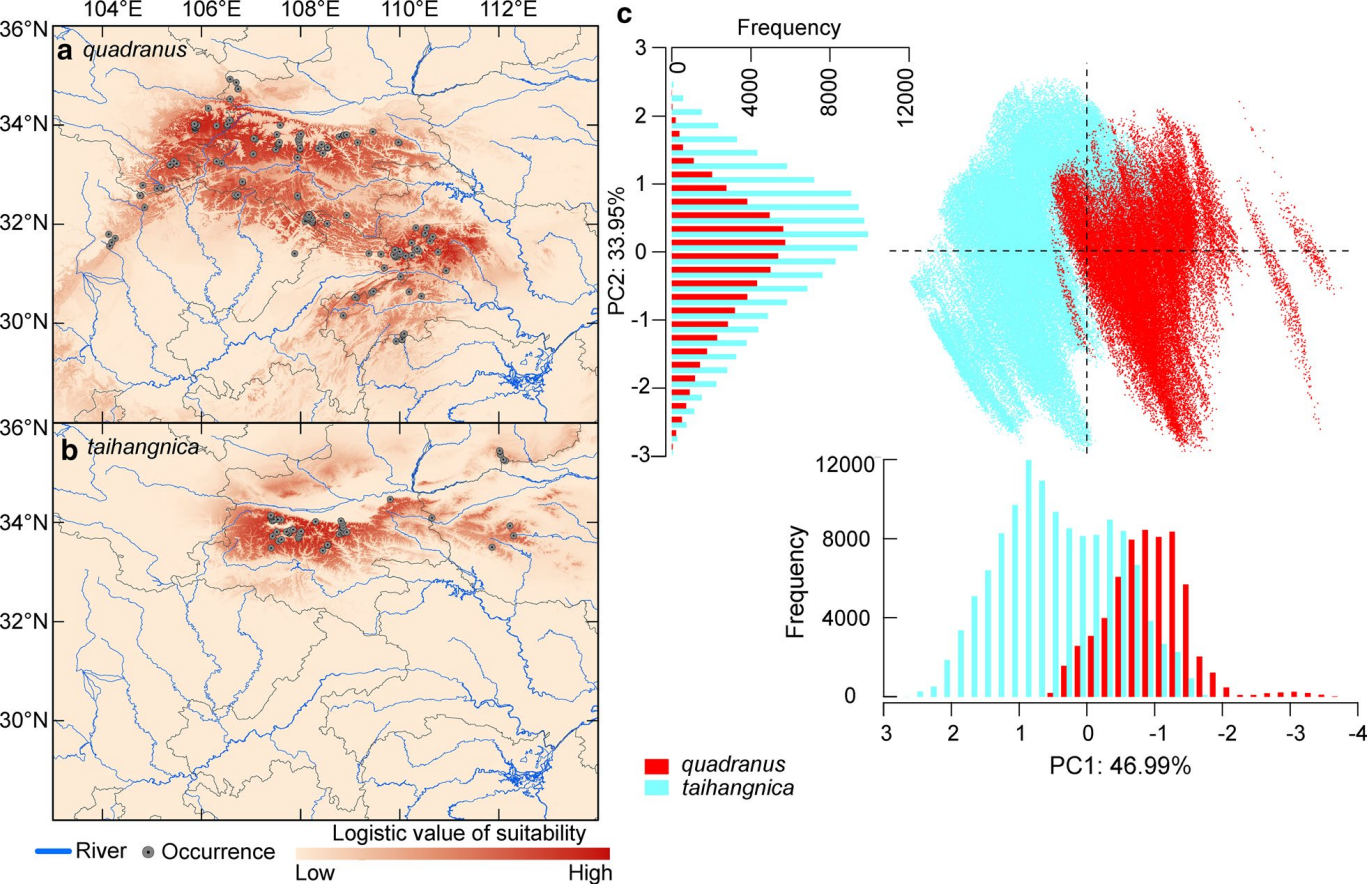
Predicted distributions, showing habitat suitability for **a** *Feirana quadranus*, **b** *F. taihangnica*, and **c** PCA plots from the ecological niche models. PCA plots are based on a logistic suitability value representing the 10th percentile training threshold of actual occurrences of each species

Considering the pattern of projected habitat suitability in G-space, areas of high suitability for *quadranus* were predicted to occur across the Qinling Mountains from west to east, and in the eastern Daba Mountains (i.e. the western-central Qinling, Micang, Daba, and Wuling Mountains; Fig. 2a). High suitability areas for *taihangnica* occurred in the central to northeastern Qinling Mountains, at scattered locations in the Funiu Mountains, and in small areas of the Zhongtiao Mountains (Fig. 2b). As described by the results of a PCA performed on ENM predictions, the first three PCs explained 93.6% of the total variance, with PC1 explaining 47.0%, PC2 explaining 34.0% and PC3 explaining 12.6%. The environmental envelopes of *quadranus* and *taihangnica* differed significantly (MANOVA: Wilk’s λ = 0.58, *P* < 0.001), with *quadranus* taking on a wider range of values of PC1 than *taihangnica*; while the predicted overlap between them was broad for PC2 (Fig. 2c).

**Fig. 3:**
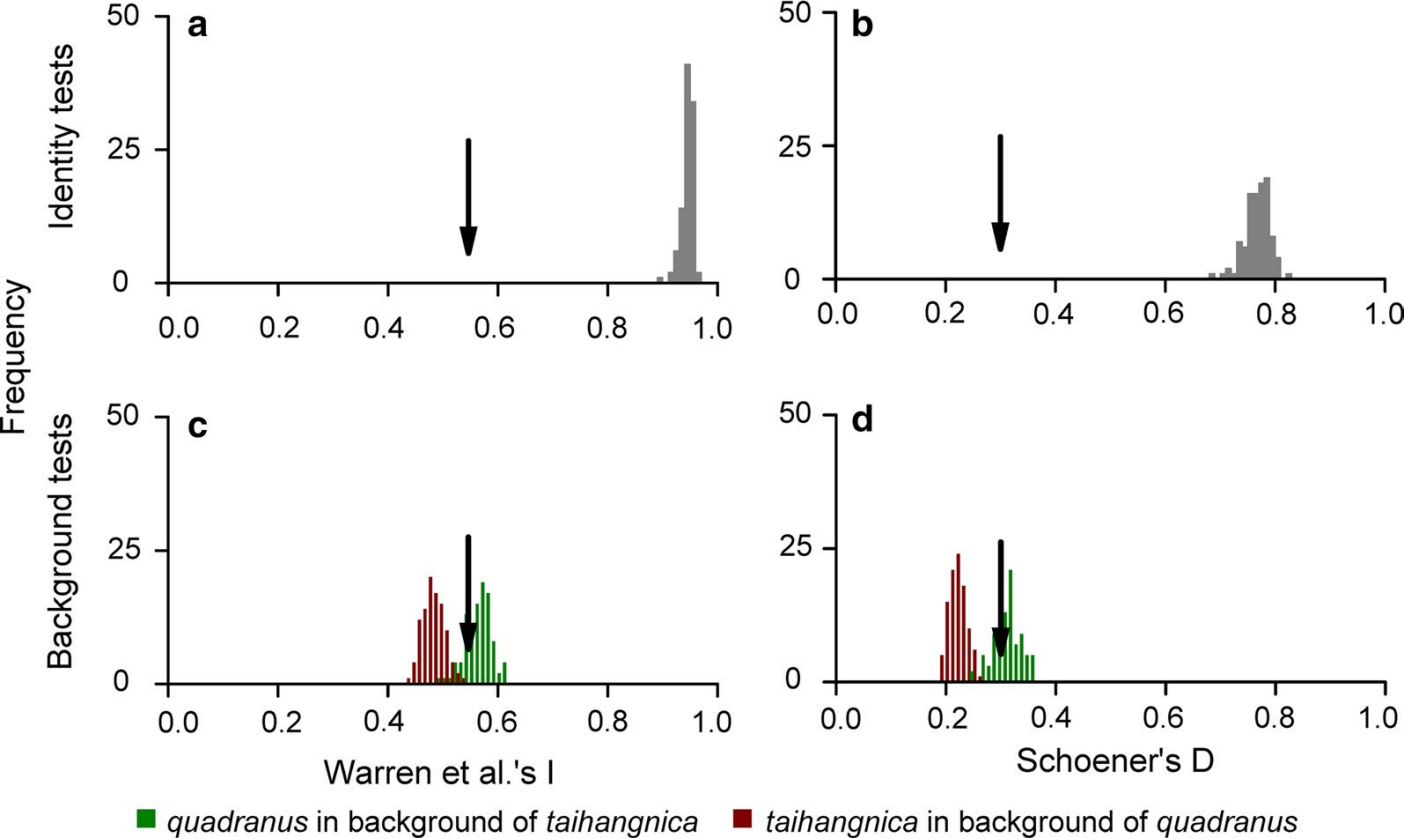
ENM-based tests of niche equivalency and background similarity. The histograms illustrate distributions of overlap scores from pseudoreplicates in niche equivalency (the upper two panels **a** & **b**) and background similarity tests (the lower two panels **c** & **d**). Arrows represent niche-overlap values

**Fig. 4:**
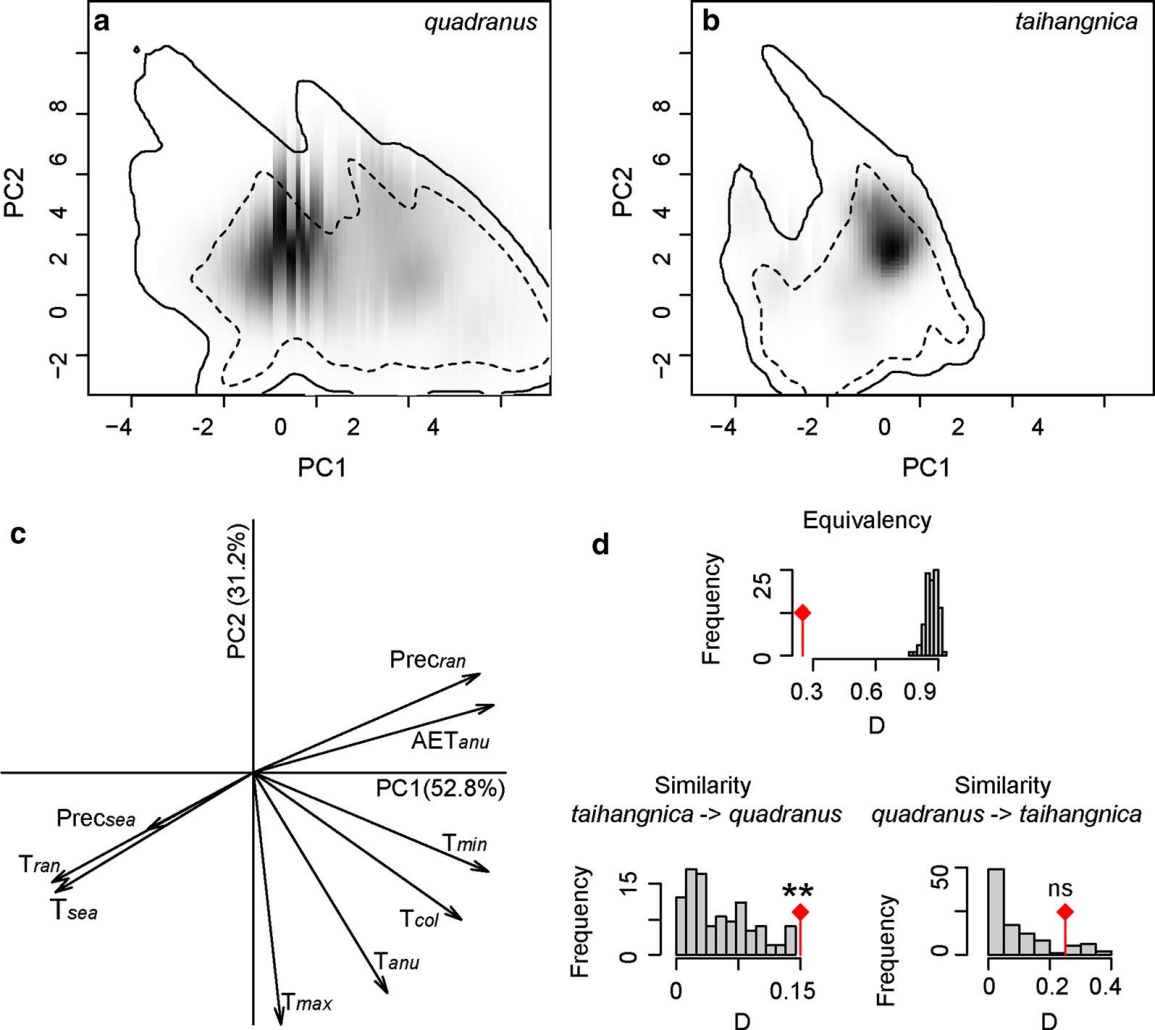
Niche of *Feirana quadranus* and *F. taihangnica* in environmental space from an ordination technique (PCA-env). In **a**, **b**, grey shading shows the density of the occurrences of species by cell. The solid and dashed contour lines illustrate, respectively, 100 and 50% of the available (background) environment. The background area is delimited by the geographic minimum convex polygon with 50-km buffer zone around occurrences of each species. **c** Represents the contribution of the environmental variables on the first two axes of the PCA and the percentage of inertia explained by the two axes. Histograms in **d** show the observed niche overlap (D) between the two species (bars with a diamond) and simulated niche overlaps (grey bars) on which tests of niche equivalency and similarity are calculated. The significance of the tests is shown (*ns* non-significant; **P < 0.05)

ENM-based niche-equivalency tests revealed that the predicted niches of *quadranus* and *taihangnica* were not equivalent, as the values of observed niche overlap fell well below the pseudoreplicated null distributions (*I* = 0.55, *D* = 0.29; both *P* < 0.01; Fig. 3a, b). Interestingly, ENM-based background similarity tests indicated greater niche conservatism between species than would be expected from their available habitats, but in only one direction. The comparison of *quadranus* to the environmental background of *taihangnica* did not deviate from the null expectation (*I* and *D*, both *P* > 0.05), while that for *taihangnica* to *quadranus* background indicated niche conservatism (*I* and *D*, both *P* < 0.01; Fig. 3c, d). Additionally, the ordination approach of PCA-env indicated that for the two species their niches in E-space overlapped little (0.25), with the first two axes explained 84.0% of the overall variance, and niche equivalency was rejected (Fig. 4a–c). Ordination null tests of niche similarity showed that niches were more similar than random in the direction of *taihangnica* to *quadranus*, while niche overlap fell within the 95% confidence limits of the null distribution, leading to non-rejection of the hypothesis of retained niche similarity for *quadranus* to *taihangnica* (Fig. 4d).

**Fig. 5:**
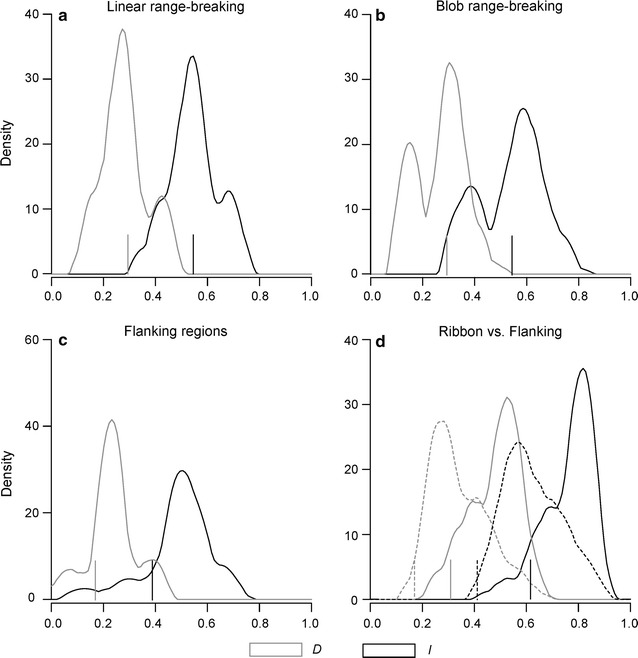
Identification of ecological determinants of parapatry from range-breaking tests. **a**, **b** Density plots indicating the distributions of *I* (black) and *D* (light grey) values from pseudo-replicates for linear and blob range-breaking analyses, respectively; **c**, of *I* and *D* values obtained from randomly generated ribbons and a comparison of flanking habitats on either side of the corresponding ribbon; and **d**, of *I* and *D* values calculated between the two flanking regions and the ribbon in each pseudoreplicate, with the dashed line for comparison of ENMs from populations with sampling comparable to *Feirana quadranus*, and the solid line for ENMs from populations with sampling comparable to *F. taihangnica*. Vertical black and light grey lines indicate *I* and *D* values from ecological niche models predictions of *F. quadranus* and *F. taihangnica* for corresponding range-breaking analyses

Linear range-breaking tests showed that the environmental divergence between *quadranus* and *taihangnica* was not different than that observed between pseudoreplicate pairs generated via random geographic fragmentation (for *I*, *P* = 0.96; for *D*, *P* = 0.92; Fig. 5a). A similar result was found when using the blob range-breaking tests (for *I*, *P* = 0.92; for *D*, *P* = 0.81, Fig. 5b). The random ribboning tests indicated that populations in flanking regions of the ribbon were no more different from one another than expected by chance (for *I*, *P* = 0.46; for *D*, *P* = 0.49; Fig. 5c). However, environmental conditions in the contact zone were more different from those in flanking regions than expected by chance for *quadranus* (*quadranus* sampling *vs.* ribbon, *P* = 0.016 and 0.048 for *I* and *D*, respectively) but not for *taihangnica* (*taihangnica* sampling *vs.* ribbon, *P* = 0.11 and 0.09 for *I* and *D*, respectively; Fig. 5d).

## Discussion

The association between climate and species’ distributions tends to be especially strong in ectotherms, owing to the close correlation of ectotherm body temperatures with ambient temperatures [4, 17]. Climatic factors (such as temperature extremes, annual mean precipitation and the seasonality of precipitation) are often used to model the geographical distributions and species richness of amphibians [31, 42]. Our results suggest that minimum temperature of the coldest month was an important factor in shaping the distribution of *Feirana quadranus*, while mean temperature of the coldest quarter was important for *F*. *taihangnica* (Additional file 3). Moreover, the degree to which ecological niches are conserved over time is continually debated in the study of niche evolution [30, 39, 43–45]. Our results revealed that the niches of *quadranus* and *taihangnica* were not equivalent. Regardless of ENM-based or ordination approach of PCA-env similarity tests, half of the comparisons suggested niche conservatism, without obvious niche divergence (Figs. 3 and 4). These findings indicate that niche conservatism probably plays a major role in the diversification of *Feirana* frogs [25, 26]. While Grinnellian niches can be conserved over short-to-moderate evolutionary time spans, the tendency to conservatism would weaken when transitioning from sister taxa to related (but not sister) species (*c.* 10^5^–10^7^ years) [43]. Our two study species diverged around 13.7 Mya [24, 29], at the point when niche conservatism theoretically should tend to break down. However, even with sufficient time to evolve different niches, the finding that *quadranus* and *taihangnica* possess highly conserved niches is not completely incongruent with the proposed tempo of niche evolution [43].

Parapatric range boundaries are generally associated with environmental gradients, in the case of closely-related species in montane regions [2, 4, 8, 9]. Both the linear and blob range-breaking tests used in this study indicated that this was not the case for *quadranus* and *taihangnica* (Fig. 5a, b). Therefore, the hypothesis that an abrupt environmental gradient formed the basis of these species’ biogeographic boundaries should be rejected [21]. Ribbon range-breaking analyses instead suggested that there was a significant environmental barrier to dispersal in the contact zone for *quadranus*, but not for *taihangnica* (Fig. 5d). Physiological constraints might limit range extension in the north, where climates are colder and drier, for *quadranus*, while adaptation to these climatic conditions may have allowed *taihangnica* to expand further north (cf. [8]). Although physical barriers can strongly influence species’ dispersal, many biogeographic boundaries could also result from ecological processes such as local adaptation and niche conservatism, or perhaps most frequently, some combination of historical and ecological processes [1, 2, 8, 9, 11]. Phylogeographic findings of much colder conditions in the central Qinling Mountains, including ice sheets on some ridges (e.g. Mt. Taibai), during the LGM, support the possible exclusion of *quadranus* from this region [25]. In contrast, populations of *taihangnica* within Henan and Shanxi provinces (Zhongtiao to the southern Taihang Mountains) represent the northern distributional limit of all frog species in the subfamily Painae [24, 27, 28]. As geographical distributions are related to thermal tolerance limits for many ectotherms [46], the barrier for *quadranus* is likely the cold, xeric conditions prevailing across the ridges of Qinling Mountains.

Previous studies of co-occurring, closely-related amphibian species have found that either unsuitable environmental conditions or interspecific competition may prevent species from extending their range boundaries [7, 9, 18]. In the Northern Hemisphere, northern range boundaries, as predicted by ENMs, should align closely with observed boundaries when abiotic factors (particularly climate) play a primary role in determining northern boundaries. However, predicted southern range boundaries (using only abiotic factors in the ENM) should extend beyond observed boundaries when interspecific interactions constrain southern boundaries [11, 18]. While ENMs should be primarily responsive to abiotic factors [10], many ENM approaches implicitly include the influences of interspecific interactions [47]. In the case of parapatric range boundaries, dissimilar factors (abiotic versus biotic factors) may limit the spatial distribution of a species to each side of the parapatric boundary [8, 18, 48, 49]. We found potential roles for both climate and interspecific competition in determining parapatric range boundaries, with the predicted northern range boundary for *quadranus* closely matching the observed range boundary and the predicted southern range boundary for *taihangnica* extending beyond the observed boundary (Fig. 2a, b). Interspecific competition between *quadranus* and *taihangnica* may have been particularly important in determining their parapatric range boundaries. Most explanations of parapatric distributions rely on negative interspecific interactions as the cause of exclusion along geographical gradients, with the more competitive species displacing the other in sympatry [18, 23, 48, 50]. *F. quadranus* has been found to be more dominant than *taihangnica* in many streams within their contact zone [29], indicating that *quadranus* most likely has higher fitness than *taihangnica* in such warm-climate regions [25, 26]. Hence, when competitive ability is asymmetric between the species, the range boundaries of the superior competitor could be determined by abiotic factors only, while interspecific competition could set the range boundaries of the inferior competitor [13, 18]. Therefore, competition with *quadranus* has probably influenced the distribution of *taihangnica* and has led to the exclusion of *taihangnica* from southern regions. However, at broad geographic scales, the spatial signature of local competitive interactions may not be detectable (cf. [51]). Future field research is critical to determine the extent of the spatial interactions and dietary overlap between these species, and how these compare to allopatric populations. Studies should also focus on large-scale components of competition, in an attempt to disentangle its precise influence on range boundaries [52].

## Conclusions

Insights into the role of abiotic versus biotic factors in setting parapatric range boundaries, which are useful in the study of ecological niches, may greatly enhance our understanding of a wide range of biological phenomena in ecology, evolution and conservation biology [4–8, 14]. Integrating our results with historical biogeographical data [24–26], we found that at a contact zone between the two parapatric frogs, a significant environmental barrier restricted *quadranus* from dispersing further north, while interspecific competition prevented the southward expansion of *taihangnica*. Our findings highlight the importance of both climate and interspecific competition in exploring ecological explanations for parapatric range boundaries between ecologically similar species. Although we studied a small group of frogs within the family Dicroglossidae, what we learned concerning the mechanisms involved in setting parapatric range boundaries can be generalized. This study contributes to our overall knowledge of the large-scale drivers of species’ presences and evolution, allowing us to make future predictions about how environmental changes may influence distributions, niche dynamics and the need for conservation [14, 23, 31, 39, 53].

## Additional files

**Additional file 1.** Distributions for occurrence records of *Feirana quadranus* and *F. taihangnica* in environmental space (annual mean temperature versus total annual precipitation).

**Additional file 2.** Filtering environmental variables.

**Additional file 3.** The jackknife test of selected variable importance for (a) *Feirana quadranus* and (b) *F. taihangnica.*

## Abbreviations

AET_*anu*_: annual actual evapotranspiration
AUC: area under the receiver operating characteristic curve
ENMs: ecological niche models
E-space: environmental space
GIS: geographic information system
G-space: geographical space
LGM: last glacial maximum
MANOVA: multivariate analysis of variance
MCP: minimum convex polygon
PCA: principal component analysis
PCs: principal components
Prec_*anu*_: annual precipitation
Prec_*sea*_: precipitation seasonality
QDM: Qinling-Daba Mountains
T_*anu*_: annual mean temperature
T_*col*_: mean temperature of the coldest quarter
T_*max*_: maximum temperature of the warmest month
T_*min*_: minimum temperature of the coldest month
T_*ran*_: mean monthly temperature range
T_*sea*_: temperature seasonality
