# Supplementary material for: Inferring ecological explanations for biogeographic boundaries of parapatric Asian mountain frogs
Source: BMC Ecol. 2018 Feb 2;18:3. doi: 10.1186/s12898-018-0160-5 (PMC5796512; doi:10.1186/s12898-018-0160-5)
Supplement: Supplementary file 1 — Additional file 1. Distributions for occurrence records of Feirana quadranus and F. taihangnica in environmental space (annual mean temperature versus total annual precipitation). [file 12898_2018_160_MOESM1_ESM.docx]

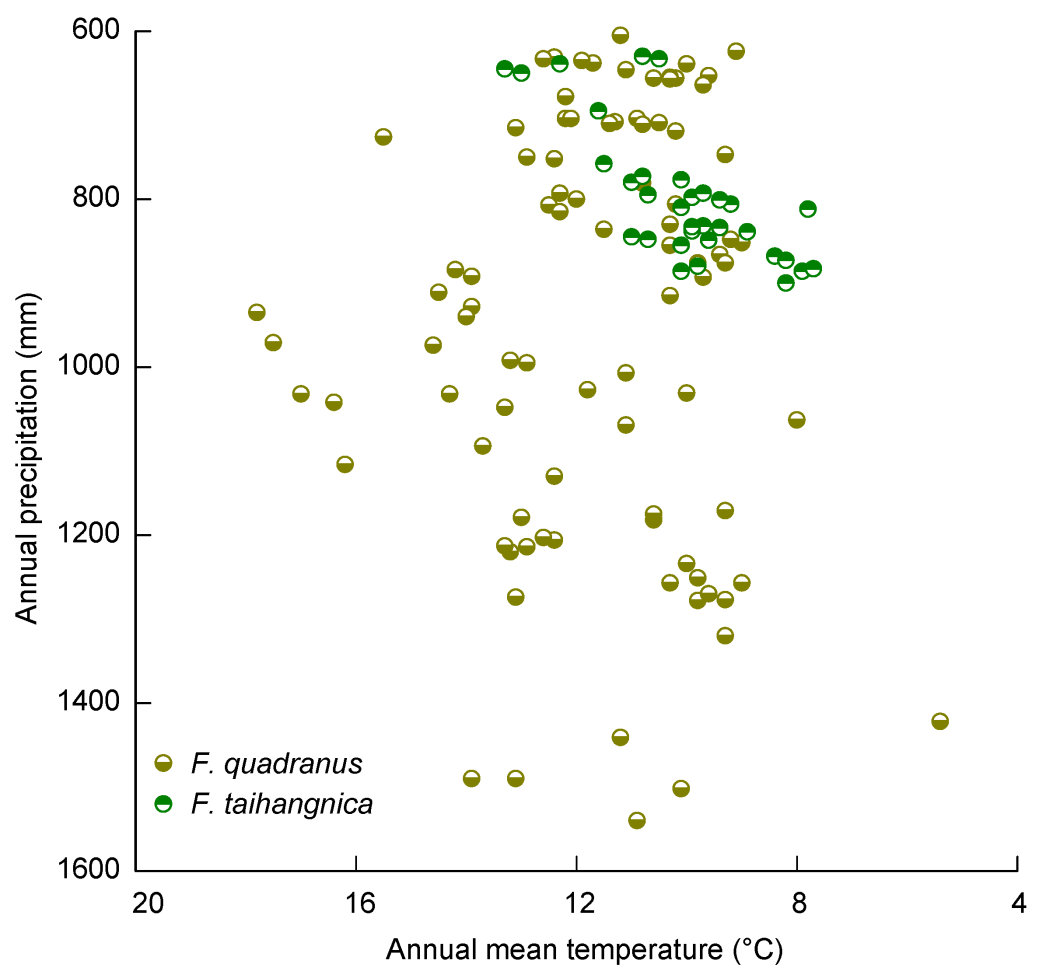


Additional file 1. Distributions for occurrence records of *Feirana quadranus* and *F. taihangnica* in environmental space (annual mean temperature versus total annual precipitation).
