# Supplementary material for: Inferring ecological explanations for biogeographic boundaries of parapatric Asian mountain frogs
Source: BMC Ecol. 2018 Feb 2;18:3. doi: 10.1186/s12898-018-0160-5 (PMC5796512; doi:10.1186/s12898-018-0160-5)
Supplement: Supplementary file 2 — Additional file 2. Filtering environmental variables. [file 12898_2018_160_MOESM2_ESM.docx]

## Additional file 2

## Filtering environmental variables

We initially compiled 28 environmental variables to characterize environmental heterogeneity across the distribution ranges for both *Feirana* *quadranus* and *F. taihangnica*. These included 19 bioclimatic variables and other nine macro-environmental variables recognized as important factors potentially shaping distributions of wildlife. The bioclimatic variables were obtained from the Worldclim 1.4 database and described estimates of annual means, degrees of seasonality, and potentially biologically limiting extremes in temperature and precipitation (Hijmans et al. 2005). These variables included: annual mean temperature (bio01), mean diurnal range (mean of monthly (max temp - min temp)) (bio02), isothermality (bio03), temperature seasonality (standard deviation * 100) (bio04), max temperature of the warmest month (bio05), min temperature of the coldest month (bio06), temperature annual range (bio07), mean temperature of the wettest quarter (bio08), mean temperature of the driest quarter (bio09), mean temperature of the warmest quarter (bio10), mean temperature of the coldest quarter (bio11), annual precipitation (bio12), precipitation of the wettest month (bio13), precipitation of the driest month (bio14), precipitation seasonality (coefficient of variation) (bio15), precipitation of the wettest quarter (bio16), precipitation of the driest quarter (bio17), precipitation of the warmest quarter (bio18), precipitation of the coldest quarter (bio19).

We obtained macro-environmental variables to consider representative soil-water balance and soil property from the Center for Sustainability and the Global Environment (<http://www.sage.wisc.edu/atlas/index.php>), and from the Consortium for Spatial Information (<http://www.cgiar-csi.org>). Growing degree days, soil organic carbon, and soil pH were obtained from the Center for Sustainability and the Global Environment. Annual actual evapotranspiration (*AET*_anu_), annual aridity index, extraterrestrial solar radiation, Priestley-Talor alpha coefficient (alpha), and annual potential evapotranspiration were obtained from the Consortium for Spatial Information. We obtained land cover data from the Global Land Cover 2000 database (GLC 2003). Due to the high levels of correlations among variables and the need for variables to be as proximal as possible (Hu and Jiang 2010, Merow et al. 2013), we filtered the initial variable set based on the results of Pearson’s correlation tests and jackknife analysis. Specially, temperature variables were removed owing to high correlations with other temperature variables (|*r*| > 0.75), and so did for precipitation variables. When using the jackknife procedure to evaluate the variable importance, the model was re-run by excluding each variable in turn; then a model was created using each variable in isolation (Phillips *et al.* 2006). Consequently, we retained nine variables that included T*_anu_* (annual mean temperature), T*_ran_* (mean monthly temperature range), T*_sea_* (temperature seasonality), T*_max_* (max temperature of the warmest month), T*_min_* (min temperature of the coldest month), T*_col_* (mean temperature of the coldest quarter), Prec*_anu_* (annual precipitation), Prec*_sea_* (precipitation seasonality) (Hijmans et al. 2005), and *AET_anu_*. All variables were at a spatial resolution of 30 arc-seconds.

# References

GLC. 2003. Global Land Cover 2000 database. European Commission, Joint Research Centre.

Hijmans, R. J., S. E. Cameron, J. L. Parra, P. G. Jones, and A. Jarvis. 2005. Very high resolution interpolated climate surfaces for global land areas. International Journal of Climatology **25**:1965-1978.

Hu, J., and Z. Jiang. 2010. Predicting the potential distribution of the endangered Przewalski’s gazelle. Journal of Zoology **282**:54-63.

Merow, C., M. J. Smith, and J. A. Silander. 2013. A practical guide to MaxEnt for modeling species’ distributions: what it does, and why inputs and settings matter. Ecography **36**:1058-1069.

Sanderson, E. W., M. Jaiteh, M. A. Levy, K. H. Redford, A. V. Wannebo, and G. Woolmer. 2002. The human footprint and the last of the wild. Bioscience **52**:891-904.
