# Supplementary material for: Inferring ecological explanations for biogeographic boundaries of parapatric Asian mountain frogs
Source: BMC Ecol. 2018 Feb 2;18:3. doi: 10.1186/s12898-018-0160-5 (PMC5796512; doi:10.1186/s12898-018-0160-5)
Supplement: Supplementary file 3 — Additional file 3. The jackknife test of selected variable importance for (a) Feirana quadranus and (b) F. taihangnica. [file 12898_2018_160_MOESM3_ESM.docx]

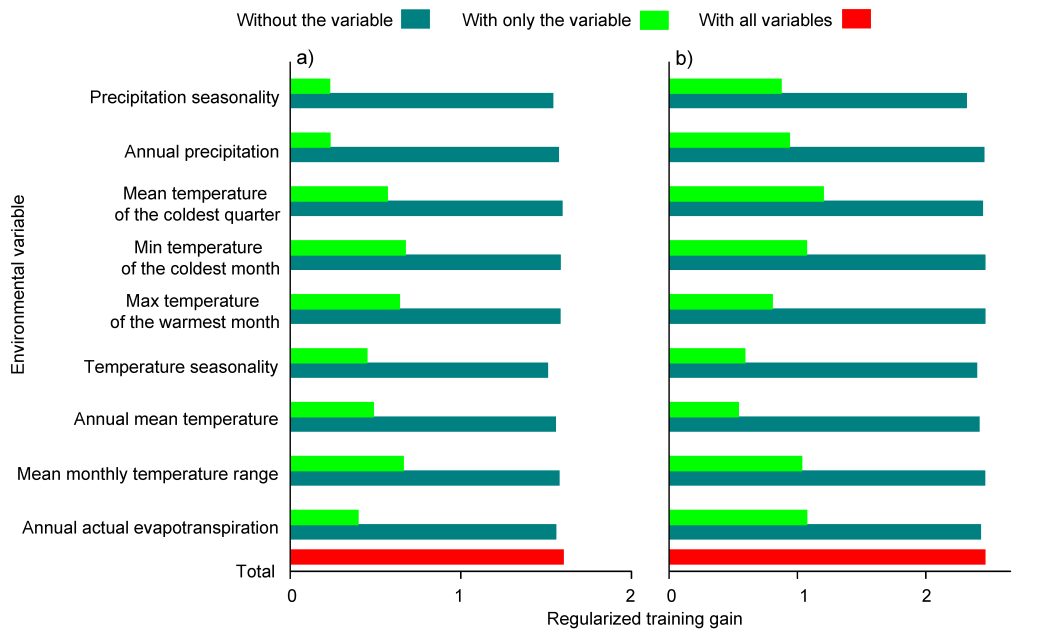


Additional file 3. The jackknife test of selected variable importance for (a) *Feirana quadranus* and (b) *F. taihangnica*.
